# The Protective Effect of Selenium on Chronic Zearalenone-Induced Reproductive System Damage in Male Mice

**DOI:** 10.3390/molecules21121687

**Published:** 2016-12-07

**Authors:** Miao Long, Shuhua Yang, Yuan Wang, Peng Li, Yi Zhang, Shuang Dong, Xinliang Chen, Jiayi Guo, Jianbin He, Zenggui Gao, Jun Wang

**Affiliations:** 1 Key Laboratory of Zoonosis of Liaoning Province, College of Animal Science & Veterinary Medicine, Shenyang Agricultural University, Shenyang 110866, China; longjlau@126.com (M.L.); yangshuhua0001@126.com (S.Y.); lm19781013@163.com (Y.W.); lipeng79625@163.com (P.L.); sihuo12345@sohu.com (Y.Z.); DongS_FOCUS@163.com (S.D.); xinliang@syau.edu.cn (X.C.); guoniuniu163@163.com (J.G.); 2 Institute of Plant Immunology, College of Plant Protection College, Shenyang Agricultural University, Shenyang 110866, China; 3 College of Animal Science and Technology, Jilin Agricultural University, Changchun 130118, China

**Keywords:** selenium, zearalenone, reproductive, testis, male mice

## Abstract

This study aims to explore the protective effect of selenium (Se) on chronic zearalenone (ZEN)-induced reproductive system damage in male mice and the possible protective molecular mechanism against this. The chronic ZEN-induced injury mouse model was established with the continuous intragastric administration of 40 mg/kg body mass (B.M.) ZEN for 28 days. Then, interventions with different doses (0.1, 0.2, and 0.4 mg/kg B.M.) of Se were conducted on mice to analyse the changes in organ indexes of epididymis and testis, antioxidant capability of testis, serum level of testosterone, sperm concentration and motility parameters, and the expression levels of apoptosis-associated genes and blood testis barrier- (BTB) related genes. Our results showed that Se could greatly improve the ZEN-induced decrease of epididymis indexes and testis indexes. Results also showed that the decrease in sperm concentration, sperm normality rate, and sperm motility parameters, including percentage of motile sperm (motile), tropism percentage (progressive) and sperm average path velocity (VAP), caused by ZEN were elevated upon administration of the higher dose (0.4 mg/kg) and intermediate dose (0.2 mg/kg) of Se. Selenium also significantly reduced the content of malondialdehyde (MDA) but enhanced the activities of antioxidant enzymes superoxide dismutase (SOD) and glutathione peroxidase (GPx) in the testis tissue. Further research demonstrated that ZEN increased the level of mRNA expression of *BCL2-associated X protein* (*Bax*) and *caspase 3* (*Casp3*), decreased the level of mRNA expression of *B cell leukemia/lymphoma 2* (*Bcl2*), *vimentin* (*Vim*) and *cadherin 2* (*Cdh2*), whereas the co-administration of Se reversed these gene expression levels. Our results indicated that high levels of Se could protect against reproductive system damage in male mice caused by ZEN and the mechanism might such be that Se improved mice antioxidant ability, inhibited reproductive cell apoptosis, and increased the decrease of BTB integrity-related genes caused by ZEN.

## 1. Introduction

Zearalenone (ZEN), a macrocyclic β-resorcyclic acid lactone, and a type of mycotoxin, is mainly produced by fungi of the *Fusarium genus. F. culmorum* [1]. Many reports revealed the risk of contamination is highest in cereal crops [2], meanwhile, silage, forage, and straw under humid conditions are also likely to contain ZEN [3]. Therefore, animals have easy access to this mycotoxin. Previous reviews report that ZEN has adverse effects on human and animal health [4]. When animals were fed a diet containing high levels of ZEN, various oestrogenic effects such as decreased fertility, increased embryolethal resorptions, reduced litter size, changed mass of adrenal, thyroid, and pituitary glands, and change in serum levels of progesterone and estradiol have been observed [4,5]. Moreover, ZEN can affect the quality of animal meat, cow milk, and chicken eggs [6]. However, the effect of ZEN is most pronounced on the reproductive system. Studies have shown that ZEN can result in breast tissue inflammation, oedematous uterus, ovarian cysts, and can cause abortion [7,8]. ZEN can also reduce male fertility. ZEN can cause testis damage to varying degrees, induce testicular atrophy and inflammation, and eventually result in decreasing sexual function with the number and quality of sperm decreased [9]. Moreover, ZEN have been found to be hepatotoxic [10], immunotoxic [11], genotoxic [12], and an enhancer of lipid peroxidation [13] in mammals. ZEN can remain and accumulate in the body for more than six months. These residues of ZEN in the body are harmful and can cause significant economic losses. Thus it can be seen that ZEN is one of the most harmful mycotoxins in the world. Therefore, how to reduce the toxicity of ZEN to animals has become a research focus. Studies demonstrated that the one of the mechanisms of ZEN toxicity is by inducing oxidative damage [14,15] and causing cell apoptosis [16,17]. Therefore, we hypothesised that the substances that have anti-oxidant and anti-apoptotic ability might alleviate the damage caused by ZEN.

Selenium (Se) is one of the elements classified within the group of micronutrients that plays an important role in the health and performance of animals [18]. Selenium participates in the protection of cells against excess reactive oxygen species, and regulation of the immune and reproductive systems [19]. Studies showed that Se has a protective role in heat-induced apoptosis and oxidative stress in mice testes [20]. When a diet is deficient in Se, it can result in the occurrence of oxidative stress and apoptosis in chicken livers [21]. However, dietary supplementation with Se in roosters can reduce apoptosis of germ cells by regulating the mRNA expressions of apoptosis- and cell cycle–related genes in the testis during spermatogenesis [22]. Studies also showed that Se has a protective effect on ischaemia-reperfusion injury in a rat testis which caused testis cell apoptosis [23] and sodium selenite supplied in the diet could effectively inhibit aflatoxin B1-induced apoptosis and cell cycle blockage in renal cells of broilers [24]. Our previous study showed that Se has strong antioxidant properties without any toxic effect on either blood parameters or serum biochemical blood markers and was able to prevent most of the alterations induced by ZEN [25]. However, it is not clear whether or not Se can protect against reproductive system damage caused by ZEN in male mice: any protective mechanism also remains to be elucidated.

The blood testis barrier (BTB) is a structural barrier between the testicular fenestrated capillaries and the interior of the seminiferous tubules, which is a physical barrier in the testis to restrict the diffusion of various endogenous and exogenous toxic chemicals in mammals [26]. Exogenous chemicals can affect the intercellular connection structure of Sertoli cells, disintegrate and destroy the structure and function of BTB, and then cause reproductive damage [27]. During this process, related proteins such as claudin, cadherin, and catenin can play important roles in the control of the BTB [28]. If these protein expressions and (or) assemblies are changed, the BTB will change and toxins then readily enter the seminiferous epithelium [29,30]. Therefore, we hypothesised that ZEN could affect the expression of BTB-related genes, such as *Cdh2* and *Vim*, then cause reproductive damage and Se could play its protective role by revising the expression of these genes. Therefore, in this study, we aimed to investigate the effects of Se on chronic ZEN-induced reproductive system damage in male mice and its possible protective mechanisms.

## 2. Results

### 2.1. Organ Indexes of Epididymis and Testis

As shown in Table 1, the epididymis, and testis, indexes were both significantly decreased in the ZEN group dosed with 40 mg/kg ZEN for 28 days compared with the control group (*p* < 0.05). The epididymis, and testis, indexes in the Se groups and the Se + ZEN groups were significantly increased compared with the ZEN group (*p* < 0.05). Compared with the ZEN group, the epididymis, and testis, indexes in the Se + ZEN groups were significantly increased (*p* < 0.05). Moreover, the epididymis, and testis, indexes in 0.2 and 0.4 mg/kg Se groups were significantly higher than that in the control group (*p* < 0.05) (Table 1).

### 2.2. Serum Level of Testosterone

As shown in Table 2, compared with the control group, the serum testosterone contents were increased in 0.1, 0.2, and 0.4 mg/kg Se groups (*p* < 0.05). The serum level of testosterone was significantly decreased in the ZEN group compared with that in the control group (*p* < 0.05). When the mice were co-treated with different concentrations of Se, the serum levels of testosterone were increased in a dose-dependent manner. The levels of testosterone in groups co-treated with Se were significantly increased compared with the ZEN group (*p* < 0.05).

### 2.3. Sperm Morphology and Deformity Rate

**Figure 1:**
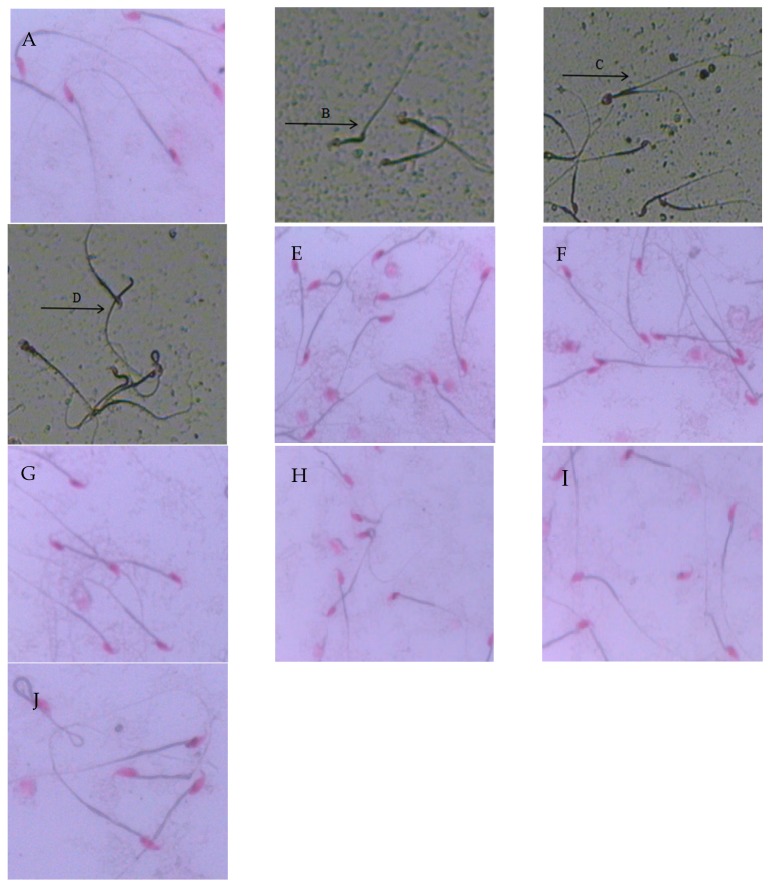
Effect of ZEN, different concentrations of selenium, and their co-treatment on morphology changes in mice sperm in mice testis (original magnification, ×400). (**A**) control group; (**B**–**D**) ZEN (40 mg/kg) group, (**E**) 0.1 mg/kg Se treatment group; (**F**) 0.2 mg/kg Se treatment group; (**G**) 0.4 mg/kg Se treatment group; (**H**) 0.1 mg/kg Se + ZEN treatment group; (**I**) 0.2 mg/kg Se + ZEN treatment group; (**J**) 0.4 mg/kg Se + ZEN treatment group. Note: (**B**): neck bending; (**C**): two tails; (**D**): no head.

As shown in Figure 1, the sperm showed normal morphologies in the control group (Figure 1A) and in the Se groups (Figure 1E–G). However, amorphous heads of sperms were seen in the ZEN group (Figure 1B–D) in forms such as: neck bending, having two tails, and having no head. Normal sperm appeared in the group co-administered with selenium yeast, although abnormal sperm occasionally appeared with a tail fold (Figure 1H–J).

As shown in Table 3, there was no significant difference in sperm deformity rates between the control group and the Se groups (*p* > 0.05); however, compared with the control group, the abnormality rate in sperm was significantly higher than that in the ZEN group (*p* < 0.05). However, when the mice were co-treated with 0.4 mg/kg Se, the sperm abnormality rate decreased significantly (*p* < 0.05).

### 2.4. Sperm Concentration and Motility Parameters

As shown in Table 3, compared with the control group, the concentration of sperm in Se groups were not significantly different (*p* > 0.05). While the concentration of sperm in the ZEN group was significantly decreased compared with that in the control group (*p* < 0.05). However, when the mice were co-administered with 0.4 mg/kg Se, the decrease in sperm concentration caused by ZEN was greater (*p* < 0.05). Compared with the control group, the sperm motility parameters, such as the percentage of motile sperm (motile), tropism percentage (progressive), and sperm average path velocity (VAP) were not significantly different in Se groups (*p* > 0.05). However, compared with the control group, these parameters were all decreased in the ZEN group (*p* < 0.05). Compared with the ZEN group, the decrease in sperm motility and progressive tropism induced by ZEN were increased in the higher dose of Se (0.4 mg/kg) group (*p* < 0.05), meanwhile, the decrease of VAP induced by ZEN were also increased in the groups dosed with Se (both at 0.2 mg/kg and 0.4 mg/kg) (*p* < 0.05).

### 2.5. Testis Antioxidant Parameters

As shown in Table 4, the contents of MDA in testis tissue of mice in the ZEN group were significantly higher than that in the control group (*p* < 0.05). Compared with the control group, the MDA levels were not significantly different in the 0.1, 0.2, and 0.4 mg/kg Se groups (*p* > 0.05). However, the MDA contents in the 0.4 mg/kg Se + ZEN group were significantly different compared with those in the ZEN group (*p* < 0.05). Compared with the control group, the activities of SOD and GPx were all increased in the 0.1, 0.2, and 0.4 mg/kg Se groups. However, compared with the control group, the activities of SOD and GPx were all decreased in the ZEN (40 mg/kg) group; however, the activities of SOD and GPx were elevated when the mice were co-administered with Se. At higher doses of Se (0.4 mg/kg), the activities of SOD and GPx were significantly different compared with those of the ZEN group (*p* < 0.05).

### 2.6. Effect on the mRNA Expression of Bax, Casp3, and Bcl2

**Figure 2:**
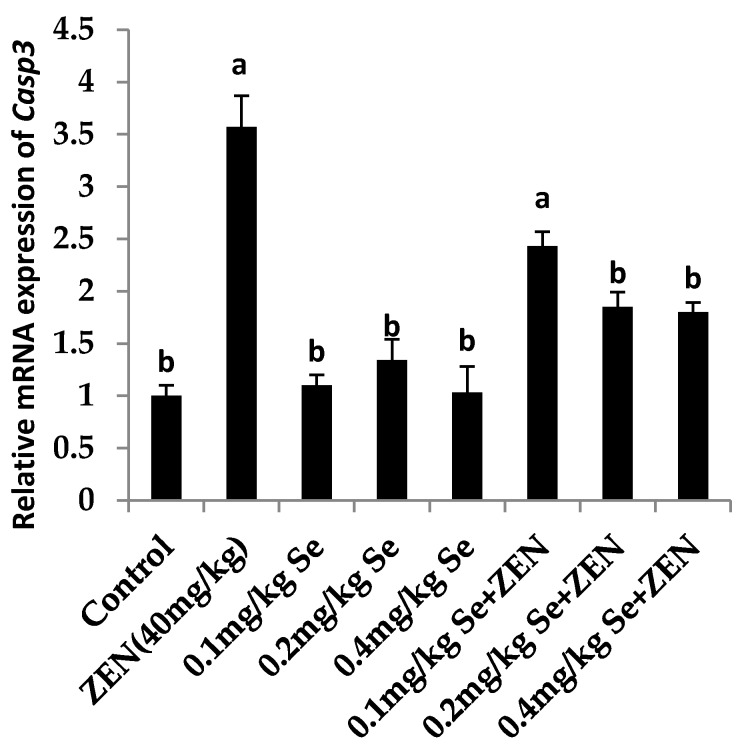
Effect of ZEN, different concentrations of Se and their co-treatment groups on the relative mRNA expression of *Casp3* in mice testicular tissue. Values are mean ± SE of twenty mice in each group. ^a,b^ Means with different letters are significantly different, *p* < 0.05. ^a^ *p* < 0.05 vs. control group, ^b^ *p* < 0.05 vs. ZEN-treated group.

**Figure 3:**
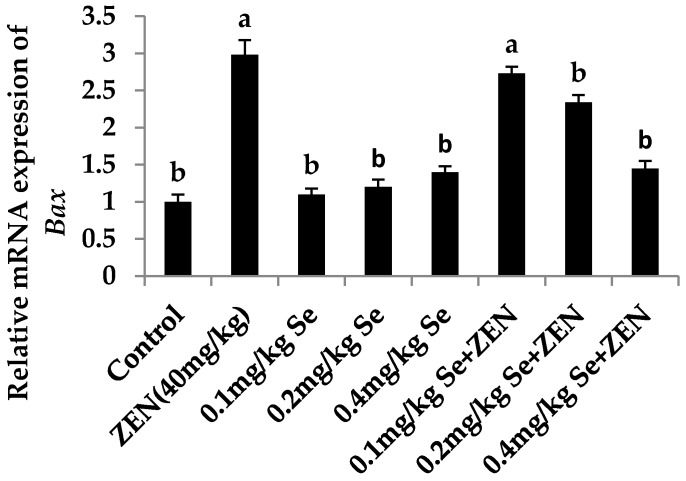
Effect of ZEN, different concentration of Se and their co-treatment on the relative mRNA expression of *Bax* in mice testicular tissue. Values are mean ± SE of twenty mice in each group. ^a,b^ Means with different letters are significantly different, *p* < 0.05. ^a^ *p* < 0.05 vs. control group, ^b^ *p* < 0.05 vs. ZEN-treated group.

**Figure 4:**
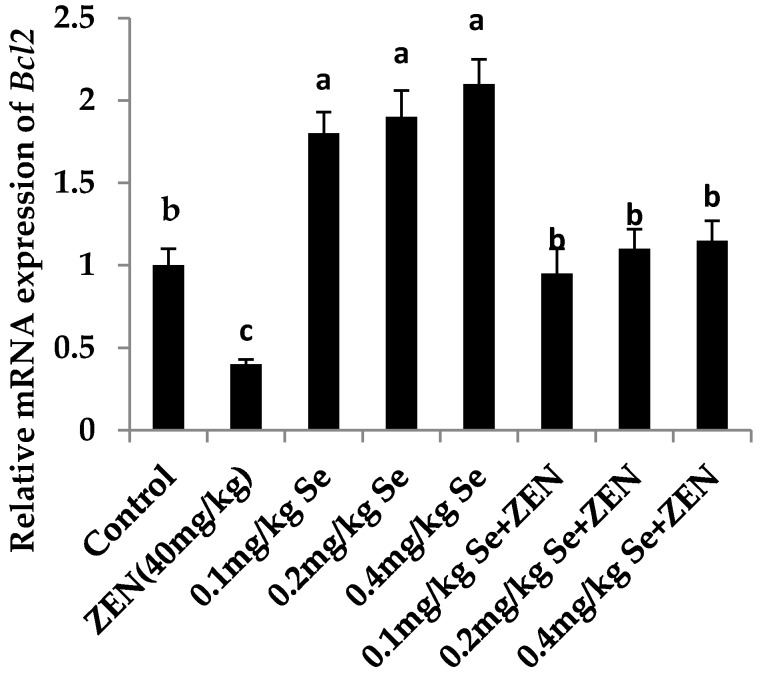
Effect of ZEN, different concentration of Se and their co-treatment on the relative mRNA expression of *Bcl2* in mice testicular tissue. Values are mean ± SE of twenty mice in each group. ^a–c^ Means with different letters are significantly different, *p* < 0.05. ^a^ *p* < 0.05 vs. control group and ZEN-treated group, ^b^ *p* < 0.05 vs. ZEN-treated group, ^c^ *p* < 0.05 vs. control group.

As shown in Figure 2 and Figure 3, compared with the control group, the mRNA expression of *Casp3* and *Bax* were increased in the ZEN group (*p* < 0.05). Although the mRNA expression of *Casp3* and *Bax* were all increased in groups with different concentrations of Se, there was no difference with that in the control group (*p* > 0.05). When the mice were co-treated with 0.4 mg/kg Se, the mRNA expressions of *Casp3* and *Bax* were decreased compared with that in the ZEN group (*p* < 0.05). The results showed that giving mice a higher level of Se could inhibit the expression of proapoptotic genes *Casp3* and *Bax* which were enhanced by ZEN. As shown in Figure 4, compared with the control group, the mRNA expressions of *Bcl2* were all increased at different concentrations of Se (*p* < 0.05). However, the mRNA expression of *Bcl2* decreased in the ZEN group (*p* < 0.05). When the mice were co-treated with 0.1, 0.2, and 0.4 mg/kg Se, respectively, the mRNA expressions of *Bcl2* were all increased whereas before they were inhibited by ZEN.

### 2.7. Effect on the mRNA Expression of Vim and Cdh2

**Figure 5:**
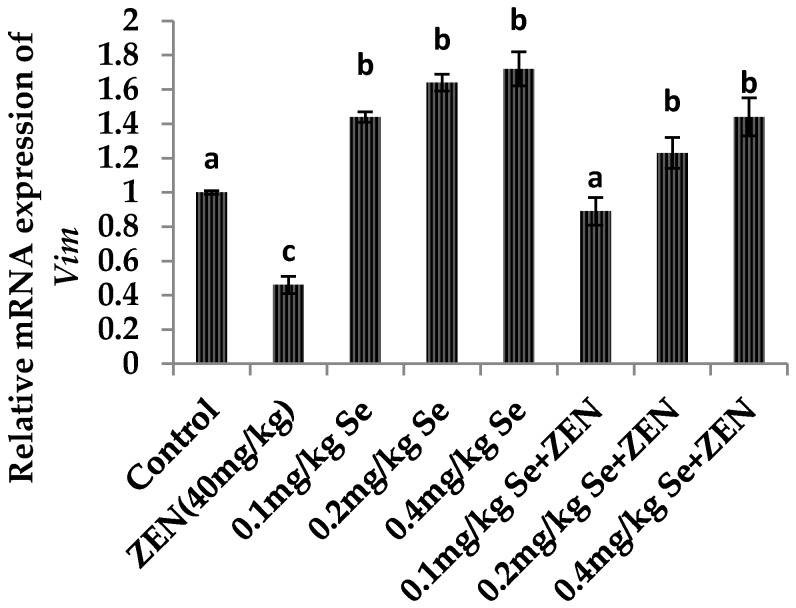
Effect of ZEN, different concentration of Se and their co-treatment on the relative mRNA expression of *Vim* in mice testicular tissue. Values are mean ± SE of twenty mice in each group. ^a–c^ Means with different letters are significantly different, *p* < 0.05. ^a^ *p* < 0.05 vs. ZEN-treated group, ^b^ *p* < 0.05 vs. control group and ZEN-treated group, ^c^ *p* < 0.05 vs. control group.

**Figure 6:**
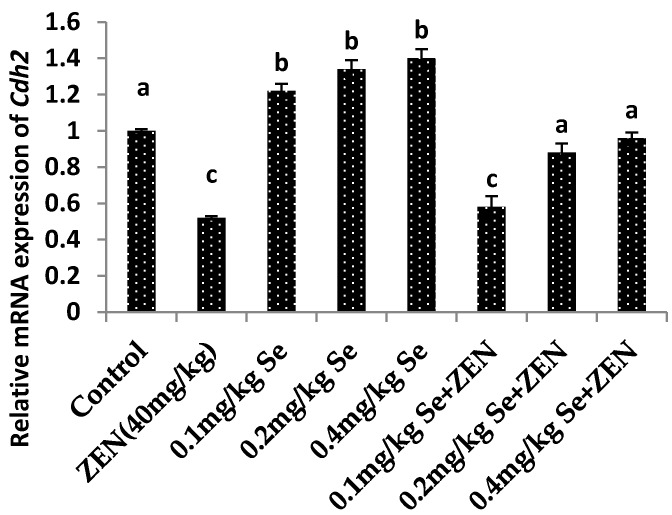
Effect of ZEN, different concentration of selenium yeast and their co-treatment on the relative mRNA expression of *Cdh2* in mice testicular tissue. Values are mean ± SE of twenty mice in each group. ^a–c^ Means with different letters are significantly different, *p* < 0.05. ^a^ *p* < 0.05 vs. ZEN-treated group, ^b^ *p* < 0.05 vs. control group and ZEN-treated group, ^c^ *p* < 0.05 vs. control group.

As shown in Figure 5 and Figure 6, compared with the control group, the mRNA expressions of *Vim* and *Cdh2* were both decreased in the ZEN group (*p* < 0.05). Meanwhile, the mRNA expression of *Vim* and *Cdh2* were all increased at different concentrations of Se: there were difference with that in the control group (*p* < 0.05). When mice were co-treated with 0.1, 0.2, and 0.4 mg/kg Se, the mRNA expressions of *Vim* and *Cdh2* were increased compared with that in the ZEN group (*p* < 0.05). The results showed that giving mice the Se could improve the gene expressions of *Vim* and *Cdh2* which were otherwise decreased by ZEN.

## 3. Discussion

In recent years, many studies have demonstrated that ZEN is harmful to animal health, and especially to reproductive function [31]. However, there are few reports of the effects of ZEN on the male reproductive system. This study extends the knowledge of ZEN toxicity on the male reproductive system.

Our results showed that after the mice were given 40 mg/kg ZEN (B.M.) for 28 days, the serum testosterone levels were decreased significantly, the concentration of sperm, the percentage of motile sperm motility (motile), tropism percentage (progressive), and sperm average path velocity (VAP) were also decreased significantly, while the rate of sperm deformity was increased. The results indicated that ZEN caused damage to the Leydig cells as testosterone is mainly secreted thereby [32]. Moreover, the results demonstrated that ZEN induced the damage of sperm in mice in vivo, which were proved by in vitro studies where ZEN was shown to reduce the hCG-stimulated testosterone synthesis of mouse Leydig cells at concentrations ranging from 10^−8^ to 10^−4^ M: this leads to a spermatogenetic disorder [33].

Studies have provided evidence that environmental toxins can induce oxidative stress, and are involved in reproductive toxicity. Furthermore, recent reports show that the toxicity of ZEN is not only due to its oestrogenicity alone [34,35]: it is demonstrated that ZEN can induce oxidative damage, which is likely to be one of the main pathways of ZEN toxicity [35,36]. Our results showed that when the mice were given 40 mg/kg ZEN (B.M.) for 28 days, the contents of MDA were increased and the activities of SOD and GPx were decreased, which results were in agreement with previous studies [37]. These results showed that ZEN could inhibit the antioxidant capacity of the body by reducing the efficacy and amount of key antioxidative enzymes.

Many studies have shown that ZEN could induce tissue cell apoptosis as a result of damage caused by oxidative stress [16]. Our results showed that ZEN up-regulated the proapoptotic *Bax* and *Casp3* mRNA expression and down-regulated the inhibiting apoptosis gene *Bcl2* mRNA expression. These results may also give the proof that ZEN can induce tissue cell apoptosis and thus make this the main mechanism of ZEN toxicity.

Selenium, as an antioxidant, can eliminate many oxygen free radicals, reduce oxidative stress damage to the body, and improve the body’s antioxidant ability and immune function [38,39]. However, whether Se can reduce the toxicity of ZEN and its effect on the reproductive system, and damage thereto, in male mice remains unclear. We used selenium yeast as an organic Se supplement because yeast is a good carrier for organic Se, and selenium yeast has both a high physiological activity and low toxicity [40]. Our results showed that when the mice were given 0.1 or 0.2 mg/kg Se for 28 days alone, the testis indexes were, to some extent, improved, which results indicated that Se had a certain function in promoting testis development. After co-administration of 0.4 mg/kg Se for 28 days, Se reduced the damage effect of ZEN on the testis and epididymis and alleviated the toxicity of ZEN on sperm. In our study, co-treatment with Se was able to improve the concentration of testosterone and oestrogen indicating that Se could protect testicular tissue. The protective mechanism might be because Se has a strong antioxidant ability to protect it from damage by lipid peroxidation, thus resulting in it inhibiting ZEN-induced testis tissue cell apoptosis. To the best of our knowledge, this is the first reported time in which Se was seen to have the ability to protect against chronic ZEN-induced reproductive system damage in male mice. When the mice were co-treated with Se, the activities of SOD and GPx were significantly increased. Previous studies showed that Se can enhance the decrease of antioxidative enzyme activities, such as SOD and GPx as induced by lithium [41] and cadmium [42]. Our results indicate that Se can elevate the decrease of antioxidant enzyme activities caused by ZEN. Se can reduce oxidative damage of testis caused by ZEN. Combined with previous studies, our results also demonstrated that Se has the ability to activate genes whose expression results in the formation of the enzymes involved in the Phase II xenobiotic metabolising pathway—the detoxification stage thereof—and slows down the synthesis of enzymes in Phase I [19,43]. At the same time, these results also explained the mechanism by which Se reduced the rate of sperm deformity, and increased the concentration of sperm and sperm motility.

The BTB is a physical barrier in the testis that restricts the diffusion of various endogenous and exogenous toxic chemicals in mammals [26]. Some proteins play important roles in the maintenance of BTB integrity. The *Vim* protein affords physical support to the BTB. The CDH2 protein has adhesion molecules and exhibits junctional adhesion, and is mainly distributed between the Sertoli cells in the basal portion: it is involved in cell adhesion and signal transduction [44]. The tightness of connection between the germ cells and the Sertoli cells is one of the important indexes used to evaluate damage to the BTB [45]. A previous study shows that ZEN could significantly reduce the mRNA expression of *Vim* and *Cdh2* in vitro [46], indicating that ZEN could destroy the BTB. We indicated that ZEN could impair the BTB by a widening of the intercellular tight junction. This research also showed that ZEN decreased the gene expression of *Vim* and *Cdh2* two key proteins affecting BTB integrity. Meanwhile, some researchers have proved that ZEN can affect the cytoskeletal structure and specific secretory functions [46] and induce apoptosis and necrosis in rat Sertoli cells in vitro [17]. Therefore, our results indicated that Se could protect the testis from damage via increasing the mRNA expression levels of the two genes, resulting in a retention of BTB integrity. However, it still needs further research to study the effect of Se on the expression of the other genes related to BTB and to examine the protein(s) related to BTB by using immunohistochemistry to illustrate the protective mechanism of Se against BTB damage.

Many studies have demonstrated that Se can protect against hepatocellular oxidative damage induced by lipopolysaccharides [47], renal damage caused by some metallic elements [48], and Aflatoxin B1 [49]. Previous studies also have demonstrated that Se appears to be mediated through its anti-apoptosis and anti-oxidative effects to protect against testis damage caused by ischaemia-reperfusion [23], cisplatin [50], electromagnetic radiation [51], and carbimazole [52]. Our results demonstrated that Se could inhibit testis tissue cells apoptosis caused by ZEN. However, which signal pathway plays the most important role in decreasing the toxic effect of ZEN by Se should be further studied in vitro. Furthermore, although the beneficial effects of Se are well known, it can also cause metabolic disorders. In the future, we will study other effects on other reproductive organ systems of male mice that may be adversely affected by treatment with Se.

## 4. Experimental Section

### 4.1. Animals

Some 160 male Kunming mice (20 ± 2 g, aged 4 weeks) were purchased from Liao Ning Chang Sheng Biotechnology Co., Ltd. (Benxi, China). The mice were bred in a room at a temperature ranging from 22 to 24 °C and the mice were subjected to 12-hour light/dark cycles at a relative humidity of between 40% to 60%. Water and the controlled diet were available on a minimum *ad libitum* basis for the mice. The mice were acclimatized for one week after transportation. The experiments have been approved by the Ethics Committee for Laboratory Animal Care (Animal Ethics Procedures and Guidelines of the People’s Republic of China) for the use of Shenyang Agricultural University, China. (Permit No. SYXK<Liao>2011-0001).

### 4.2. Chemicals

ZEN was obtained from Sigma (St. Louis, MO, USA), which was prepared at a concentration of 200 mg/mL as a stock solution by using diethyl sulphoxide and the solution was stored at −20 °C. The working solution was obtained by dispensing the stock solution into the 0.9 % physiological saline, and the concentration of ZEN was 1 mg/mL: at this concentration, the DMSO content was 0.5%. Selenium yeast was obtained from the Angel Yeast Co. Ltd. (Wuhan, China; with a Se content of 2000 mg/kg). The kits used for measuring GPx, SOD, and MDA activities were obtained from Nanjing Jiancheng Bioengineering Institute (Nanjing, China); the SYBR green RT-PCR kit (Takara, Japan), and DAPI (Sigma Aldrich, St. Louis, MO, USA) were also used. The primers for *Casp3*, *Bax*, *Bcl2*, and *Actb* were synthesised and purified by Sangon Biotech (Shanghai, China); moreover, the preservation solution of RNA samples and the kits for total animal RNA extraction were obtained from Sangon Biotech Co., Ltd., while the kits for Reversion Aid First Strand cDNA Synthesis were purchased from MBI Fermentas (Burlington, ON, Canada). The Iodine [^125^I] Testosterone Radioimmunoassay Kit was purchased from Beijing North Biological Technology Research Institute Co., Ltd. (Beijing, China).

### 4.3. Experimental Design and Treatment

The control group (*n* = 20): the mice were dosed by intragastric administration with physiological saline, daily, for 28 days.

The ZEN group (*n* = 20): the mice were dosed by intragastric administration with a 40 mg/kg dose of ZEN (B.M.) (40 mg/kg—8 % of LD_50_), which was based on other preliminary experiments [53,54], daily for 28 days.

The 0.1 mg/kg Se group (*n* = 20): the mice were given 0.1 mg/kg Se B.M. by intragastric administration with a 0.05 g dose of selenium yeast (containing 2 g/kg Se) diluted with physiological saline, daily, for 28 days.

The 0.2 mg/kg Se group (*n* = 20): the mice were given 0.2 mg/kg Se B.W. by intragastric administration with a 0.1 g dose of selenium yeast (containing 2 g/kg Se) diluted with physiological saline, daily, for 28 days.

The 0.4 mg/kg Se group (*n* = 20): the mice were given 0.2 mg/kg Se B.W. by intragastric administration with a 0.2 g dose of selenium yeast (containing 2 g/kg Se) diluted with physiological saline, daily, for 28 days.

The 0.1 mg/kg Se + ZEN group (*n* = 20): after given 0.1 mg/kg Se B.W. by intragastric administration with a 0.05 g dose of selenium yeast at 9:00 a.m., the mice were given a 40 mg/kg dose of ZEN (B.W.) in the same way at 15:00 p.m., daily, for 28 days.

The 0.2 mg/kg Se + ZEN group (*n* = 20): after given 0.2 mg/kg Se B.W. by intragastric administration with a 0.1 g dose of selenium yeast at 9:00 a.m., at 9:00 a.m., the mice were given a 40 mg/kg dose of ZEN (B.W.) in the same way at 15:00 p.m., daily, for 28 days.

The 0.4 mg/kg Se + ZEN group (*n* = 20): after given 0.4 mg/kg Se B.W. by intragastric administration with a 0.2 g dose of selenium yeast at 9:00 a.m., the mice were given a 40 mg/kg dose of ZEN (B.W.) in the same way at 15:00 p.m., daily, for 28 days.

At 24 h after the last pre-treatment, the mice were weighed and blood was collected from the endocanthion. The mice were then sacrificed by cervical dislocation. From this sample, the serum was separated by centrifugation and stored at −80 °C until assayed. From each group we randomly selected five mice to excise and weigh both testes and epididymides. Then, the other mice testes tissues were weighed and then stored at −80 °C for further use in the experiments, and the other mice epididymis were immediately put into 2 mL 0.9% saline at 37 °C to extract sperm after being isolated from each mouse.

### 4.4. Parameters

#### 4.4.1. Epididymis, and Testis, Indexes

After the last administration (and a delay of approximately 24 h), the body, the paired epididymides, and paired testes were weighed. The organ index was given by:

Organ index = (organ mass (g)/body mass (g)) × 100 %
(1)

#### 4.4.2. Serum Testosterone Levels in Mice

The radioimmunoassay method was used to detect serum testosterone levels. The details of the determination procedures followed the manufacturer’s instructions for the Iodine [^125^I] Testosterone Radioimmunoassay Kit.

#### 4.4.3. Sperm Deformity Rate

A drop of fresh semen and a drop of 1% eosin solution were mixed on a glass slide and covered with a cover-slip. After 30 s, the semen was observed under an optical microscope at ×400 magnification. The sperm morphology was observed according to the dead sperm which were stained red and the rate of sperm abnormality was calculated.

#### 4.4.4. Sperm Concentration and Motility

After dilution of the semen, the sperm concentration was calculated by blood cell counting plate method. The computer assisted sperm analysis system (CASA) was used to analyse sperm motility and activity mode such as motility, progressive tropism, and average path velocity (VAP).

#### 4.4.5. Antioxidant

In the experiment, the content of MDA, and the activities of GPx and SOD were examined to analyse the oxidant levels of the mice testis. SOD, MDA, and GPx assay kits were used to carry out the analysis. The details of all determination procedures followed the manufacturer’s instructions for the commercial kits.

### 4.5. Gene Expression

The total RNA of the testis was extracted using TRIzol reagent. Then, the purity of the total RNA was measured via the quotient for OD at 260/280 nm. The mRNA was then reverse transcribed into cDNA using a MBI Fermentas PrimeScript RT reagent kit. The cDNA was adopted as the template for quantitative RT-PCR analysis. An ABI 7500 real-time PCR system and the SYBR Green PCR Kit were used to conduct real-time PCR. Each sample was measured in triplicate.

For the qRT-PCR reactions, 2 μL product of cDNA, 0.4 μL reverse primer, 0.4 μL forward primer, 10 μL 2 × SYBR^®^ Premix Ex Taq™, 6.8 μL of RNase-freewater, and 0.4 μL ROX Reference Dye II (50×) were used. The conditions under which the PCR reaction was conducted included: at the initial stage, denaturing at 95 °C for 5 min, and then denaturing at 95 °C for 10 s, annealing at 60 °C for 5 s, and extension at 60 °C for 34 s. The amount of template was measured based on the standard curve for such a quantitative analysis. The gene expressions of *Casp3*, *Bcl2*, and *Bax* were analysed, and *Actb* was used as a housekeeping gene. The primers of *Casp3*, *Bcl2*, *Bax*, *Vim*, *Cdh2*, and *Actb* are listed in Table 5. The results were analysed by using the 2^−ΔΔCT^ assay.

### 4.6. Statistical Analysis

Results were presented as the mean ± standard error (X ± SE). Firstly, one-way ANOVA was used to assess the significance of differences among mean values. Afterwards, a Student- Newman-Keuls (SNK) *post hoc* test, or the least significant difference (LSD) multiple, was used via pair-wise comparisons. In addition, SPSS 17 software (IBM, Almon, NY, USA) was used to carry out all statistical tests. Mean values were proven to be significantly different at *p* < 0.05.

## 5. Conclusions

In summary, Se can improve the ZEN-induced decrease of the concentration of sperm, the sperm motility parameters, and reduce the rate of occurrence of deformities in sperm. The protection mechanism might be such that Se could inhibit the oxidative stress and apoptosis of reproductive-related cells induced by ZEN and Se could play its protective role by increasing the gene expression of *Vim* and *Cdh2*.

## Figures and Tables

**Table 1:** Effect of Se on organ indexes of epididymis and testis in mice induced by ZEN.

Group	Organ Indexes of Epididymis (%)	Organ Indexes of Testis (%)
Control	0.108 ± 0.006 ^b^	0.393 ± 0.012 ^b^
ZEN (40 mg/kg)	0.073 ± 0.004 ^a^	0.305 ± 0.006 ^a^
0.1 mg/kg Se	0.112 ± 0.003 ^b^	0.418 ± 0.009 ^b^
0.2 mg/kg Se	0.120 ± 0.008 ^c^	0.433 ± 0.010 ^c^
0.4 mg/kg Se	0.125 ± 0.005 ^c^	0.440 ± 0.007 ^c^
0.1 mg/kg Se + ZEN	0.093 ± 0.006 ^b^	0.410 ± 0.005 ^b^
0.2 mg/kg Se + ZEN	0.095 ± 0.005 ^b^	0.416 ± 0.006 ^b^
0.4 mg/kg Se + ZEN	0.097 ± 0.008 ^b^	0.420 ± 0.008 ^b^

^a–c^ Means within the column with different letters are significantly different, *p* < 0.05. Epididymis indexes: paired epididymides weight (g)/body weight (g) × 100%; Testis indexes: paired testes weight (g)/body weight (g) × 100%. ^a^ *p* < 0.05 vs. control group, ^b^ *p* < 0.05 vs. ZEN-treated group, ^c^ *p* < 0.05 vs. control group and ZEN-treated group.

**Table 2:** Effect of Se on serum level of testosterone in mice induced by ZEN.

Group	Serum Level of Testosterone (ng/mL)
Control	7.443 ± 1.064 ^c^
ZEN (40 mg/kg)	0.153 ± 0.008 ^a^
0.1 mg/kg Se	9.973 ± 1.107 ^b^
0.2 mg/kg Se	10.404 ± 1.139 ^b^
0.4 mg/kg Se	11.967 ± 1.007 ^b^
0.1 mg/kg Se + ZEN	0.684 ± 0.011 ^b^
0.2 mg/kg Se + ZEN	0.933 ± 0.005 ^b^
0.4 mg/kg Se + ZEN	1.475 ± 0.007 ^b^

^a,b,c^ Means within the column with different letters are significantly different, *p* < 0.05. ^a^ *p* < 0.05 vs. control group, ^b^ *p* < 0.05 vs. control group and ZEN-treated group, ^c^ *p* < 0.05 vs. ZEN-treated group.

**Table 3:** Effect of Se on sperm deformity rate, sperm concentration and sperm motility in mice induced by ZEN.

Group	Sperm Deformity Rate (%)	Sperm Number (million/mL)	Motile (%)	Progressive (%)	VAP (μm/s)
Control	12.22 ± 2.11 ^a^	21.49 ± 1.58 ^a^	90.41 ± 2.17 ^a^	15.33 ± 2.77 ^a^	75.42 ± 4.34 ^a^
ZEN (40 mg/kg)	24.56 ± 4.20 ^b^	10.06 ± 0.55 ^b^	38.52 ± 1.30 ^b^	6.78 ± 1.49 ^b^	48.72 ± 3.33 ^b^
0.1 mg/kg Se	13.58 ± 3.66 ^a^	22.42 ± 1.80 ^a^	90.36 ± 1.85 ^a^	15.03 ± 1.74 ^a^	74.32 ± 2.86 ^a^
0.2 mg/kg Se	13.23 ± 3.26 ^a^	21.51 ± 1.88 ^a^	91.54 ± 1.80 ^a^	15.65 ± 1.88 ^a^	76.73 ± 2.56 ^a^
0.4 mg/kg Se	12.56 ± 3.41 ^a^	22.98 ± 1.61 ^a^	92.30 ± 1.63 ^a^	16.38 ± 1.93 ^a^	80.16 ± 2.21 ^a^
0.1 mg/kg Se + ZEN	20.84 ± 3.52 ^b^	12.33 ± 0.91 ^b^	81.56 ± 1.39 ^c^	6.66 ± 1.04 ^b^	54.33 ± 1.59 ^b^
0.2 mg/kg Se + ZEN	20.46 ± 3.09 ^b^	16.06 ± 1.23 ^c^	82.82 ± 1.31 ^c^	7.28 ± 1.61 ^b^	70.43 ± 1.69 ^a^
0.4 mg/kg Se + ZEN	13.45 ± 2.52 ^a^	20.15 ± 1.43 ^a^	88.36 ± 1.53 ^a^	14.46 ± 1.47 ^a^	72.40 ± 1.58 ^a^

^a–c^ Means within the column with different letters are significantly different, *p* < 0.05. ^a^ *p* < 0.05 vs. ZEN-treated group, ^b^ *p* < 0.05 vs. control group, ^c^ *p* < 0.05 vs. control group and ZEN-treated group. Motile: The mice motile sperm percentage; Progressive: The mice sperm tropism percentage; VAP: average of path velocity.

**Table 4:** Effect of Se on testis antioxidant parameters in mice induced by ZEN.

Group	MDA (nmol/mgprot)	SOD (U/mgprot)	GPx (mg/gprot)
control	0.437 ± 0.067 ^b^	55.149 ± 0.788 ^b^	47.118 ± 3.878 ^b^
ZEN (40 mg/kg)	1.255 ± 0.039 ^c^	35.006 ± 0.450 ^c^	29.112 ± 1.80 ^c^
0.1 mg/kg Se	0.510 ± 0.057 ^b^	65.429 ± 0.800 ^a^	55.460 ± 3.45 ^b^
0.2 mg/kg Se	0.526 ± 0.068 ^b^	67.713 ± 1.088 ^a^	57.421 ± 3.088 ^b^
0.4 mg/kg Se	0.683 ± 0.037 ^b^	68.998 ± 2.310 ^a^	75.460 ± 1.230 ^a^
0.1 mg/kg Se + ZEN	1.182 ± 0.021 ^c^	56.630 ± 1.319 ^b^	29.604 ± 3.319 ^c^
0.2 mg/kg Se + ZEN	0.981 ± 0.038 ^c^	59.006 ± 1.031 ^b^	32.421 ± 3.301 ^b^
0.4 mg/kg Se + ZEN	0.710 ± 0.020 ^b^	60.050 ± 1.43 ^b^	45.625 ± 3.432 ^b^

^a–c^ Means within the column with different letters are significantly different, *p* < 0.05. ^a^ *p* < 0.05 vs. control group and ZEN-treated group, ^b^ *p* < 0.05 vs. ZEN-treated group, ^c^ *p* < 0.05 vs. control group. MDA: malondialdehyde; GPx: glutathione peroxidase; SOD: superoxide dismutase.

**Table 5:** Primers for real-time PCR analyses.

Gene	Primer	Primer Sequences (5’-3’)	Product Size/bp	Accession No.
*Casp3*	Forward	CTGACTGGAAAGCCGAAACTC	189 bp	NM_009810.2
	Reverse	CGACCCGTCCTTTGAATTTCT		
*Bax*	Forward	CAGGATGCGTCCACCAAGAA	197 bp	NM_007527.3
	Reverse	GCAAAGTAGAAGAGGGCAACCAC		
*Bcl2*	Forward	GCTACCGTCGTCGTGACTTCGC	147 bp	NM_177410.2
	Reverse	CCCCACCGAACTCAAAGAAGG		
*Vim*	Forward	GATCAGCTCACCAACGACAA	120 bp	NM_011701.4
	Reverse	GCTTTCGGCTTCCTCTCTCT		
*Cdh2*	Forward	AGGACCCTTTCCTCAAGAGC	117 bp	AB008811.1
	Reverse	ATAATGAAGATGCCCGTTGG		
*Actb*	Forward	CTGTCCCTGTATGCCTCTG	221 bp	BC_138614.1
	Reverse	TTGATGTCACGCACGATT		
